# Sociodemographic Variations in Women’s Reports of Discussions With Clinicians About Breast Density

**DOI:** 10.1001/jamanetworkopen.2023.44850

**Published:** 2023-11-27

**Authors:** Nancy R. Kressin, Jolie B. Wormwood, Tracy A. Battaglia, Priscilla J. Slanetz, Christine M. Gunn

**Affiliations:** 1 Section of General Internal Medicine, Department of Medicine, Boston University Chobanian & Avedisian School of Medicine, Boston, Massachusetts; 2 Department of Psychology, University of New Hampshire, Durham; 3 Department of Radiology, Boston University Chobanian & Avedisian School of Medicine, Boston, Massachusetts; 4 The Dartmouth Institute for Health Policy and Clinical Practice, Geisel School of Medicine, Dartmouth College, Hanover, New Hampshire; 5 Dartmouth Cancer Center, Geisel School of Medicine, Dartmouth College, Hanover, New Hampshire

## Abstract

**Importance:**

Breast density notifications advise women to discuss breast density with their clinicians, yet little is known about such discussions.

**Objectives:**

To examine the content of women’s reports of breast density discussions with clinicians and identify variations by women’s sociodemographic characteristics (age, income, state legislation status, race and ethnicity, and literacy level).

**Design, Setting, and Participants:**

This US nationwide, population-based, random-digit dial telephone survey study was conducted from July 1, 2019, to April 30, 2020, among 2306 women aged 40 to 76 years with no history of breast cancer who underwent mammography in the prior 2 years and had heard the term *dense breasts* or *breast density*. Results were analyzed from a subsample of 770 women reporting a conversation about breast density with their clinician after their last mammographic screening. Statistical analysis was conducted in April and July 2023.

**Main Outcomes and Measures:**

Survey questions inquired whether women’s clinicians had asked about breast cancer risk or their worries or concerns about breast density, had discussed mammography results or other options for breast cancer screening or their future risk of breast cancer, as well as the extent to which the clinician answered questions about breast density.

**Results:**

Of the 770 women (358 [47%] aged 50-64 years; 47 Asian [6%], 125 Hispanic [16%], 204 non-Hispanic Black [27%], 317 non-Hispanic White [41%], and 77 other race and ethnicity [10%]) whose results were analyzed, most reported that their clinicians asked questions about breast cancer risk (88% [670 of 766]), discussed mammography results (94% [724 of 768]), and answered patient questions about breast density (81% [614 of 761]); fewer women reported that clinicians had asked about worries or concerns about breast density (69% [524 of 764]), future risk of breast cancer (64% [489 of 764]), or other options for breast cancer screening (61% [459 of 756]). Women’s reports of conversations varied significantly by race and ethnicity; non-Hispanic Black women reported being asked questions about breast cancer risk more often than non-Hispanic White women (odds ratio [OR], 2.08 [95% CI, 1.05-4.10]; *P* = .04). Asian women less often reported being asked about their worries or concerns (OR, 0.42 [95% CI, 0.20-0.86]; *P* = .02), and Hispanic and Asian women less often reported having their questions about breast density answered completely or mostly (Asian: OR, 0.28 [95% CI, 0.13-0.62]; *P* = .002; Hispanic: OR, 0.48 [95% CI, 0.27-0.87]; *P* = .02). Women with low literacy were less likely than women with high literacy to report being asked about worries or concerns about breast density (OR, 0.64 [95% CI, 0.43-0.96]; *P* = .03), that mammography results were discussed with them (OR, 0.32 [95% CI, 0.16-0.63]; *P* = .001), or that their questions about breast density were answered completely or mostly (OR, 0.51 [95% CI, 0.32-0.81]; *P* = .004).

**Conclusions and Relevance:**

In this survey study, although most women reported that their clinicians counselled them about breast density, the unaddressed worries or concerns and unanswered questions, especially among Hispanic and Asian women and those with low literacy, highlighted areas where discussions could be improved.

## Key Points

**Question:**

Do patient-clinician discussions about breast density vary by women’s sociodemographic characteristics?

**Findings:**

In this telephone survey study of 770 women, most women reported that clinicians asked questions about breast cancer risk (88%), discussed mammography results (94%), and answered patient questions about breast density (81%); fewer women indicated that clinicians asked about worries or concerns about breast density (69%), future breast cancer risk (64%), or other screening options (61%). Non-Hispanic Black women were asked about breast cancer risk more often, and Hispanic and Asian women as well as those with low literacy were less likely to have questions or worries or concerns addressed.

**Meaning:**

This study suggests that unaddressed worries or concerns and unanswered questions among Hispanic and Asian women as well as those with low literacy are areas for improvement.

## Teaser

This survey study examines the content of women’s reports of breast density discussions with clinicians as well as variations in these discussions by women’s sociodemographic characteristics.

## Introduction

Dense breast tissue increases the risk of breast cancer and reduces the sensitivity of screening mammography.^1,2^ Recognizing these risks, dense breast notifications (DBNs) are mandated by legislation in 38 US states and the District of Columbia, with nationwide US government notification starting in 2024. Dense breast notifications accompany mammography results and provide information about breast density aimed at increasing women’s understanding of associated risks. Most notifications recommend that women with dense breasts discuss their personal risk with their physicians to foster more informed decisions about future and supplemental breast cancer screening.^3^

However, prior research shows that less than half of women in the general population have conversations with their clinicians about breast density,^4,5^ and little is known about the content of the conversations that do occur. By understanding women’s experiences of such conversations, including whether they confer relevant information about breast cancer risk and screening options or address patients’ questions and concerns—all necessary components for shared decision-making—we can identify strengths and address potential deficits. As prior research showed that women who belong to minoritized racial and ethic groups and with lower literacy were often less knowledgeable or aware of breast density’s risks,^4^ we hypothesized that women’s conversations with clinicians might also vary similarly.

## Methods

Using a telephone survey, we assessed women’s reported experiences of conversations with their clinicians about breast density. Survey content was based on existing literature, prior studies,^6,7,8,9^ and the Health Belief Model.^10,11^ The research was deemed exempt in the category of survey research by the Boston University Medical Campus institutional review board; however, a consent statement read to prospective participants provided options to decline or continue. This study followed the American Association for Public Opinion Research (AAPOR) reporting guideline.

### Participant Sampling and Recruitment

We conducted a cross-sectional, national, random-digit dial telephone survey study using a sampling approach detailed previously.^4^ In brief, structured telephone surveys from the survey firm SSRS included questions within the SSRS Omnibus survey, a national, weekly, dual-frame bilingual random-digit dial telephone survey using landlines and cell phones, conducted in English and Spanish. Our questions were included in the Omnibus survey from July 1, 2019, to April 30, 2020. We identified a representative sample of US women 40 to 76 years of age meeting the following eligibility criteria: (1) had undergone mammography within the prior 2 years, (2) had no personal history of breast cancer, and (3) had heard the term *dense breasts* or *breast density*. Eligible participants were also invited to participate using a prescreened callback sample from prior Omnibus waves to increase participation of Hispanic, Black, and Asian women; women with less than a high school education; and those living in less populous nonnotification states. A third group of eligible participants were recruited from a sample specifically modeled by SSRS to reach Asian American women. The Figure delineates the yield and AAPOR cooperation rates (proportion of completed interviews received out of qualified respondents who were selected to complete the interview^12^) from each sampling approach; the overall cooperation rate was 85% (2306 of 2718). This analysis was conducted among the 770 of 2306 women in the sample (33%) who reported having had a conversation about breast density with their clinician after their last mammographic screening.

**Figure.**
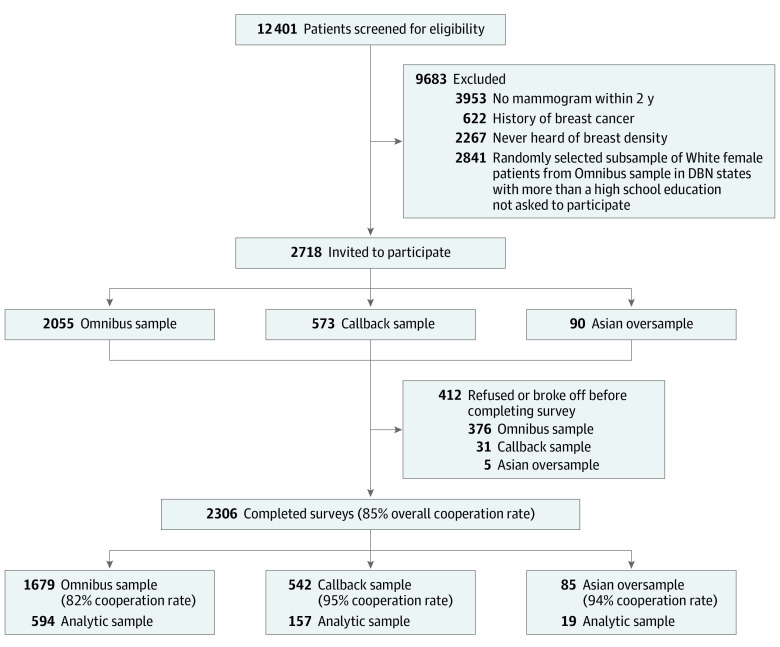
Cohort Derivation DBN indicates dense breast notification.

### Measures

We queried participants about their most recent discussion with their clinician regarding breast density. As detailed in the eAppendix in Supplement 1, we asked whether the clinician had (1) asked questions about breast cancer risk (family history or prior biopsies), (2) asked about worries or concerns about breast density, (3) discussed mammography results, (4) discussed other options for breast cancer screening (eg, magnetic resonance imaging or ultrasonography), or (5) discussed the woman’s future risk of breast cancer (all yes or no responses; answers from respondents who were unsure or declined to answer were designated missing). Then, we asked about the extent to which their clinician had answered their questions about breast density (completely, mostly, somewhat, a little, or not at all). Given our focus on the most desirable outcome, this variable was dichotomized for analyses into completely or mostly and somewhat, a little, or not at all.

We assessed whether or not respondents lived in a state mandating DBNs, sociodemographic characteristics including age (40-49, 50-64, and ≥65 years), educational level (high school or less, some college, and college or more), income (<$50 000, $50 000-$99 999, and ≥$100 000), and race and ethnicity (Asian, Hispanic or Latino, non-Hispanic Black, non-Hispanic White, and other [Native American or American Indian or Alaska Native, Native Hawaiian and Other Pacific Islander, mixed, and other (as indicated by the respondent)]). We assessed literacy, defining low literacy as either having less than a high school education or reporting sometimes, often, or always needing assistance to complete forms using the validated Single Item Literacy Screener.^13^ To assess and control for breast cancer risk factors as covariates, we asked each woman whether she had a first-degree relative with a diagnosis of breast cancer or had ever had a breast biopsy.

### Statistical Analysis

Statistical analysis was conducted in April and July 2023. We first conducted bivariate χ^2^ analyses assessing associations between each dependent variable and each of the sociodemographic variables listed. We then conducted separate multivariable binary logistic regression analyses, evaluating associations among each dependent variable and all of the sociodemographic variables and breast cancer risk factors simultaneously. We excluded educational level from multivariable models due to its inclusion in the health literacy measure and collinearity with income. All analyses were conducted with the statistical analysis software IBM SPSS Statistics, version 27 (IBM Corp). Statistical significance was defined at 2-tailed α = .05. Post hoc comparisons in bivariate analyses were Bonferroni corrected for multiple comparisons.

## Results

Among the analytic sample (770 of 2306 [33%]), most women (630 [82%]) lived in states mandating DBNs (Table 1). Approximately half the women were aged 50 to 64 years (358 [47%]), with 188 (24%) aged 40 to 49 years and 224 (29%) aged 65 years or older. A total of 348 of 721 women (48%) had incomes less than $50 000, 211 of 721 (29%) had incomes between $50 000 and $99 999, and 162 of 721 (23%) had incomes of $100 000 or higher. A total of 174 of 766 women (23%) had a high school education or less, 226 of 766 (30%) had some college education, and 366 of 766 (48%) had a college education or greater; 156 (20%) had low literacy. A total of 47 women (6%) were Asian, 125 (16%) were Hispanic, 204 (27%) were non-Hispanic Black, 317 (41%) were non-Hispanic White, and 77 (10%) were of other race and ethnicity. Women in the analytic sample were more likely than those not in the analytic sample to reside in a DBN state (82% [630 of 770] vs 75% [1152 of 1536]; χ^2^_1_ = 13.58; *P* < .001), be younger (age 40-49 years, 24% [188 of 770] vs 17% [261 of 1536]; χ^2^_2_ = 19.26; *P* < .001), have a lower annual income level (<$50 000, 48% [348 of 721] vs 43% [614 of 1437]; χ^2^_2_ = 6.37; *P* = .04), to have a lower level of literacy (20% [156 of 770] vs 15% [232 of 1536]; χ^2^_1_ = 9.74; *P* = .002), and to have a prior breast biopsy (30% [229 of 768] vs 22% [330 of 1521]; χ^2^_1_ = 18.24; *P* < .001). They were also more likely than those not in the analytic sample to be of other race (10% [77 of 770] vs 5% [83 of 1535]) and less likely to be non-Hispanic White (41% [317 of 770] vs 48% [741 of 1535]) (χ^2^_4_ = 26.15; *P* < .001).

**Table 1.** Sample Characteristics

Characteristic	No. (%)		*P* value
	Had conversation (analysis sample) (n = 770)	Did not have conversation (n = 1536)	
In DBN state	630 (82)^a^	1152 (75)^a^	<.001
Age group, y			
40-49	188 (24)^a^	261 (17)^a^	<.001
50-64	358 (47)	749 (49)	
≥65	224 (29)^a^	526 (34)^a^	
Income, $			
<50 000	348 (48)^a^	614 (43)^a^	.04
50 000-99 999	211 (29)^a^	482 (34)^a^	
≥100 000	162 (23)^a^	341 (24)^a^	
Missing	49	99	
Educational level			
High school or less	174 (23)	324 (21)	.61
Some college	226 (30)	448 (29)	
College or more	366 (48)	762 (50)	
Missing, No.	4	2	
Race and ethnicity			
Asian	47 (6)	121 (8)	<.001
Hispanic	125 (16)	213 (14)	
Non-Hispanic Black	204 (27)	377 (25)	
Non-Hispanic White (reference)	317 (41)^a^	741 (48)^a^	
Other^b^	77 (10)^a^	83 (5)^a^	
Missing, No.	0	1	
Literacy			
Low	156 (20)^a^	232 (15)^a^	.002
High	614 (80)^a^	1304 (85)^a^	
Individual breast cancer risk factors			
Family history of breast cancer	143 (19)	267 (18)	.49
Missing, No.	6	13	
Prior breast biopsy	229 (30)^a^	330 (22)^a^	<.001
Missing, No.	2	15	

Abbreviation: DBN, dense breast notification.

^a^ Individual comparisons are significantly different across groups. The *P* value is for the χ^2^ analysis comparing each categorical variable by conversation status.

^b^ Includes Native American or American Indian or Alaska Native, Native Hawaiian and Other Pacific Islander, mixed, and other (as indicated by the respondent).

Overall, 88% of women (670 of 766) said that their clinician had asked questions about breast cancer risk (Table 2). In bivariate analyses, women with a college degree or higher reported being asked questions about their risk more often (90% [326 of 363]) than women with a high school education or less (84% [145 of 173]) (*P* < .05). Non-Hispanic Black women more often reported being asked questions about breast cancer risk (92% [187 of 204]) than Hispanic women (82% [101 of 124]) (*P* < .05). Similarly, multivariable results revealed that non-Hispanic Black women were 2.08 times more likely to report questions about breast cancer risk than non-Hispanic White women (odds ratio [OR], 2.08 [95% CI, 1.05-4.10]; *P* = .04) (Table 3). In multivariable analyses, questions about risk were also more often reported by women with a family history of breast cancer (OR, 2.86 [95% CI, 1.27-6.43]; *P* = .01) or prior biopsy (OR, 1.79 [95% CI, 1.01-3.17]; *P* = .045).

**Table 2.** Women’s Recall of Experiences Discussing Breast Density With Clinicians Among Women Who Did Have a Discussion About Breast Density After Last Mammographic Screening (N = 770): Bivariate Results

Overall, No./total No. (%)	DBN state, No./total No. (%)		Age, No./total No. (%)			Income, No./total No. (%)			Educational level, No./total No. (%)			Race and ethnicity, No./total No. (%)					Literacy level, No./total No. (%)	
	Yes	No	40-49 y	50-64 y	≥65 y	<$50 000	$50 000-$99 999	≥$100 000	≤HS	Some college	≥College	NH White	NH Black	Hispanic	Asian	Other	High	Low
**When you last discussed breast density with your physician or other clinician, did they also ask questions about your breast cancer risk?**																		
670/766 (88)	546/628 (87)	124/138 (90)	164/186 (88)	314/357 (88)	192/223 (86)	296/346 (86)	188/211 (89)	144/160 (90)	145/173 (84)^a^	196/226 (87)	326/363 (90)	275/315 (87)	187/204 (92)^b^	101/124 (82)	39/46 (85)	68/77 (88)	542/612 (89)	128/154 (83)
**When you last discussed breast density with your physician or other clinician, did they also ask about any worries or concerns you might have about your breast density?**																		
524/764 (69)	428/626 (68)	96/138 (70)	137/187 (73)^c^	258/354 (73)^c^	129/223 (58)	224/345 (65)^d^	155/209 (74)	109/161 (68)	112/172 (65)	151/224 (67)	257/364 (71)	221/316 (70)^e^	145/203 (71)^e^	79/124 (64)	24/46 (52)	55/75 (73)^e^	432/609 (71)^f^	92/155 (59)
**When you last discussed breast density with your physician or other clinician, did they also discuss your mammography results with you?**																		
724/768 (94)	592/629 (94)	132/139 (95)	180/188 (96)	329/356 (92)	215/224 (96)	321/347 (93)	197/210 (94)	157/162 (97)	166/174 (95)	208/226 (92)	346/364 (95)	299/316 (95)^b^	197/204 (97)^b^	109/124 (88)	43/47 (92)	76/77 (99)^b^^,^^e^	588/613 (96)^f^	136/155 (88)
**When you last discussed breast density with your physician or other clinician, did they also discuss other options for breast cancer screening?**																		
459/756 (61)	380/620 (61)	79/136 (58)	109/186 (59)	231/353 (65)^c^	119/217 (55)	206/342 (60)	116/205 (57)	102/161 (63)	98/171 (57)	129/221 (58)	229/360 (64)	187/311 (60)	121/199 (61)	72/123 (59)	34/47 (72)	45/76 (59)	362/606 (60)	97/150 (65)
**When you last discussed breast density with your physician or other clinician, did they also discuss your future risk of getting breast cancer?**																		
489/764 (64)	403/627 (64)	86/137 (63)	127/188 (68)	230/356 (65)	132/220 (60)	223/346 (65)	133/208 (64)	102/162 (63)	109/173 (63)	142/224 (63)	234/363 (65)	204/314 (65)	122/204 (60)	81/123 (66 )	31/47 (66)	51/76 (67)	393/610 (64)	96/154 (62)
**To what extent did your physician or clinician answer your questions about breast density? (% completely or mostly)**																		
614/761 (81)	496/622 (80)	118/139 (85)	150/187 (80)	286/353 (81)	178/221 (81)	271/344 (79)	174/209 (83)	130/159 (82)	137/171 (80)	175/224 (78)	299/362 (83)	270/315 (86)	160/203 (79)^g^	89/120 (74)^g^	33/47 (70)^g^	62/76 (82)	505/609 (83)^f^	109/152 (72)

Abbreviations: DBN, dense breast notification; HS, high school; NH, non-Hispanic.

^a^ Significantly different from college or more after Bonferroni correction for multiple comparisons.

^b^ Significantly different from Hispanic after Bonferroni correction for multiple comparisons.

^c^ Significantly different from 65 years or older after Bonferroni correction for multiple comparisons.

^d^ Significantly different from $50 000 to $99 999 after Bonferroni correction for multiple comparisons.

^e^ Significantly different from Asian after Bonferroni correction for multiple comparisons.

^f^ Significantly different from low literacy after Bonferroni correction for multiple comparisons.

^g^ Significantly different from NH White after Bonferroni correction for multiple comparisons.

**Table 3.** Women’s Reports of Experiences Discussing Breast Density With Clinicians: Multivariable Results

Characteristic	Ask questions about your breast cancer risk? (n = 709)	Ask about any worries or concerns you might have about your breast density? (n = 707)	Discuss your mammography results with you? (n = 711)	Discuss other options for breast cancer screening? (n = 700)	Discuss your future risk of getting breast cancer? (n = 708)	Answer your questions about breast density (completely or mostly)? (n = 705)
Omnibus χ^2^ value	29.17	35.46	39.96	19.89	9.85	23.74
Omnibus *P* value	.004^a^	<.001^a^	<.001^a^	.07	.63	.02^a^
−2LL	502.66	850.58	284.65	922.32	915.53	670.59
Constant	8.44	2.27	60.37	1.11	1.73	6.74
DBN state, OR (95% CI)	0.81 (0.41-1.61)	1.11 (0.69-1.79)	1.15 (0.44-3.00)	1.12 (0.72-1.76)	1.04 (0.66-1.63)	1.00 (0.55-1.84)
Race and ethnicity, OR (95% CI)						
Asian	0.67 (0.26-1.73)	0.42 (0.20-0.86)^a^	0.36 (0.10-1.28)	1.52 (0.72-3.18)	1.00 (0.49-2.04)	0.28 (0.13-0.62)^a^
Hispanic	0.82 (0.43-1.56)	0.76 (0.46-1.26)	0.37 (0.16-0.88)^a^	0.93 (0.58-1.51)	1.11 (0.68-1.82)	0.48 (0.27-0.87)^a^
Non-Hispanic Black	2.08 (1.05-4.10)^a^	1.11 (0.71-1.74)	1.48 (0.56-3.93)	1.14 (0.75-1.73)	0.90 (0.60-1.37)	0.65 (0.38-1.11)
Non-Hispanic White	[Reference]	[Reference]	[Reference]	[Reference]	[Reference]	[Reference]
Other	1.43 (0.58-3.50)	1.17 (0.60-2.25)	NA	1.14 (0.64-2.05)	1.46 (0.78-2.72)	0.78 (0.36-1.68)
Literacy, OR (95% CI)						
Low	0.66 (0.38-1.13)	0.64 (0.43-0.96)^a^	0.32 (0.16-0.63)^a^	1.32 (0.88-1.98)	0.91 (0.61-1.36)	0.51 (0.32-0.81)^a^
High	[Reference]	[Reference]	[Reference]	[Reference]	[Reference]	[Reference]
Age, OR (95% CI)						
<50 y	[Reference]	[Reference]	[Reference]	[Reference]	[Reference]	[Reference]
50-64 y	1.07 (0.60-1.91)	1.04 (0.68-1.58)	0.57 (0.24-1.34)	1.27 (0.87-1.86)	0.89 (0.60-1.32)	1.13 (0.70-1.82)
≥65 y	0.86 (0.45-1.65)	0.49 (0.31-0.77)^a^	1.42 (0.50-4.04)	0.79 (0.51-1.22)	0.70 (0.45-1.09)	0.99 (0.58-1.70)
Income, OR (95% CI)						
<$50 000	0.62 (0.32-1.21)	1.10 (0.70-1.71)	0.41 (0.14-1.18)	0.88 (0.58-1.35)	1.11 (0.72-1.69)	0.93 (0.54-1.59)
$50 000-$99 999	0.82 (0.40-1.66)	1.50 (0.94-2.40)	0.45 (0.15-1.36)	0.77 (0.50-1.19)	1.06 (0.68-1.65)	1.11 (0.63-1.95)
≥$100 000	[Reference]	[Reference]	[Reference]	[Reference]	[Reference]	[Reference]
Family history of breast cancer, OR (95% CI)	2.86 (1.27-6.43)^a^	1.17 (0.60-2.25)	0.70 (0.31-1.58)	1.41 (0.93-2.15)	1.52 (0.99-2.34)	1.10 (0.66-1.84)
Prior biopsy, OR (95% CI)	1.79 (1.01-3.17)^a^	1.09 (0.76-1.57)	1.04 (0.50-2.14)	1.54 (1.09-2.18)^a^	1.05 (0.75-1.48)	0.94 (0.62-1.43)

Abbreviations: DBN, dense breast notification; NA, not applicable; OR, odds ratio; –2LL, –2 log likelihood.

^a^ Statistically significant.

Overall, 69% of women (524 of 764) indicated their clinician had asked about worries or concerns about breast density. In bivariate analyses, women who were aged 65 years or older reported receiving fewer questions about worries or concerns (58% [129 of 223]) compared with younger women (aged 50-64 years, 73% [258 of 354]; <50 years, 73% [137 of 187]; *P* < .05) (Table 2), and this difference held in multivariable analyses (OR, 0.49 [95% CI, 0.31-0.77]; *P* = .002) (Table 3). In bivariate analyses, women with incomes lower than $50 000 were less likely to report questions about worries or concerns (65% [224 of 345]) than women with incomes of $50 000 to $99 999 (74% [155 of 209]) (Table 2), although there were no differences by income in the multivariable analyses (<$50 000 vs ≥$100 000: OR, 1.10 [95% CI, 0.70-1.71]; *P* = .69; $50 000-$99 999 vs ≥$100 000: OR, 1.50 [95% CI, 0.94-2.40]; *P* = .09) (Table 3). In bivariate analyses, Asian women were less likely to report receiving such questions (52% [24 of 46]) than non-Hispanic White women (70% [221 of 316]), non-Hispanic Black women (71% [145 of 203]), and women of other racial and ethnic groups (73% [55 of 75]) (*P* < .05) (Table 2). This pattern held in multivariable analyses: the odds of an Asian woman reporting such questions were less than half the odds of a non-Hispanic White woman (OR, 0.42 [95% CI, 0.20-0.86]; *P* = .02) (Table 3). Women with low literacy also reported questions about worries or concerns less often (59% [92 of 155]) than women with high literacy (71% [432 of 609]) in both bivariate (*P* < .05) (Table 2) and multivariable analyses (OR, 0.64 [95% CI, 0.43-0.96]; *P* = .03) (Table 3).

Overall, 94% of women (724 of 768) said their clinician discussed their mammography results. However, bivariate analyses revealed this was less common among women with low literacy (88% [136 of 155]) compared with women with high literacy (96% [588 of 613]) (*P* < .05) (Table 2). This association remained significant in multivariable analyses (OR, 0.32 [95% CI, 0.16-0.63]; *P* = .001) (Table 3). Hispanic women were less likely (88% [109 of 124]) than non-Hispanic White women (95% [299 of 316]), non-Hispanic Black women (97% [197 of 204]), and women of other racial or ethnic groups (99% [76 of 77]) to report such discussions (*P* < .05) (Table 2). Similarly, in multivariable analyses, the odds of a Hispanic woman reporting discussing her mammography results with a clinician were significantly lower than the odds for a non-Hispanic White woman (OR, 0.37 [95% CI, 0.16-0.88; *P* = .02) (Table 3).

Overall, 61% of women (459 of 756) indicated their clinician had discussed other options for breast cancer screening. In bivariate analyses, women aged 50 to 64 years were more likely (65% [231 of 353]) to report such discussions than women aged 65 years or older (55% [119 of 217]) (*P* < .05) (Table 2), although we did not see significant differences by age in multivariable analyses (50-64 years vs <50 years: OR, 1.27 [95% CI, 0.87-1.86]; *P* = .22; ≥65 years vs <50 years: OR, 0.79 [95% CI, 0.51-1.22]; *P* = .28) (Table 3). In multivariable analyses, reports of discussions about other screening options were more likely among women with prior biopsy (OR, 1.54 [95% CI, 1.09-2.18]; *P* = .01).

Overall, 64% of women (489 of 764) indicated that their clinician discussed future risk of breast cancer. We found no differences by any sociodemographic characteristics or breast cancer risk factors in either bivariate or multivariable analyses (Table 2 and Table 3).

Overall, 81% of women (614 of 761) indicated their clinician had mostly or completely answered their questions about breast density, but such reports varied by women’s race and ethnicity. In bivariate analyses, non-Hispanic White women were more likely (86% [270 of 315]) than non-Hispanic Black women (79% [160 of 203]), Hispanic women (74% [89 of 120]), or Asian women (70% [33 of 47]) to say their clinician had answered their questions (*P* < .05) (Table 2). This pattern generally held in multivariable analyses, such that Hispanic (OR, 0.48 [95% CI, 0.27-0.87]; *P* = .02) and Asian women (OR, 0.28 [95% CI, 0.13-0.62]; *P* = .002) were less likely than non-Hispanic White women to indicate their clinician had answered all of their questions about breast density (Table 3). Women with low literacy were also less likely to indicate their questions were answered (72% [109 of 152]) than women with high literacy (83% [505 of 609]) in bivariate analyses (*P* < .05) (Table 2), and this association held in multivariable analyses (OR, 0.51 [95% CI, 0.32-0.81]; *P* = .004) (Table 3).

## Discussion

As one of the first studies, to our knowledge, to examine women’s reports of the frequency and content of clinician-patient breast density discussions, we examined whether or how such discussions might vary by women’s sociodemographic characteristics. Overall, only approximately one-third of women surveyed reported such a conversation, similar to other studies.^4,5^ Findings newly revealed that these women were more likely to be members of historically marginalized groups, including lower income or literacy levels or women of minority racial and ethnic groups. Among women reporting such conversations, most indicated that clinicians asked about breast cancer risk, discussed mammography results, and answered questions about breast density. However, women less frequently reported that clinicians had asked about worries or concerns about breast density or discussed future risk of breast cancer or other options for breast cancer screening.

Women’s reports of these discussions varied significantly by race and ethnicity. Non-Hispanic Black women (a group at higher risk for breast cancer mortality) more often reported being queried about breast cancer risk, but Hispanic and Asian women were less likely to report being asked about worries or concerns or having their questions about breast density answered completely or mostly. Although our data do not provide an explanation for this observed difference, clinician behavior may be influenced by the evidence of elevated breast cancer mortality among young Black women in particular.^14^ The single prior study we located regarding the content of women’s discussions with physicians about breast density found no differences between the proportion of non-Hispanic White and non-Hispanic Black women who reported talking with their physicians about breast density notifications,^5^ but those results did not characterize the discussions’ content.

Women’s reported breast density discussions with clinicians varied significantly with literacy level. Women with low literacy less frequently reported being asked about worries or concerns about breast density or that their mammography results were discussed with them and less frequently felt their questions were answered completely or mostly. This finding highlights a mismatch between patients’ informational needs and material shared by clinicians; one possibility is that clinicians limit the information offered to patients when discussing breast density due to assumptions about the ability of patients with low literacy to understand information about screening or its consequences,^15^ but our data do not allow us to examine this. Perhaps the information given was less comprehensible to women with low literacy, as prior research shows that some clinicians struggle with providing understandable information to such patients.^16^ Regardless, our findings suggest that women with low literacy want more or different information to meet their needs.

Clinician questioning and counseling did not vary by state DBN legislation status, suggesting that the legislation either was associated with broad or no nationwide outcomes or that other clinician education has generally been associated with care. We are unable to determine this with our data given the single time point of measurement. Clinician questioning and counseling almost never varied by women’s age or income. As might be expected, women with risk factors (eg, family history or prior benign breast biopsy result) were more likely to report being asked questions about breast cancer risk; the latter group was also more likely to indicate that their clinician discussed other options for breast cancer screening. Women with a family history of breast cancer could have a heightened sensitivity to the topic, which could in turn have increased recall of discussions of breast cancer risk, although we were unable to examine this possibility directly.

Our findings suggest that when discussions about breast density do occur, over half of women’s clinicians are addressing relevant related topics. Shortfalls in addressing breast density may be a function of clinicians feeling ill prepared,^7,17,18,19,20^ so additional education about the risks of breast density may support clinician knowledge and effective counseling on this topic. Inconsistent insurance coverage of supplemental screening and the absence of a widely agreed-on evidence base^21^ or guidelines regarding supplemental screening to guide counseling at the time these data were collected may have inhibited such counseling and the incorporation of recommended shared decision-making, which could improve the quality of conversations.^22^ The limited clarity regarding supplemental screening may undergird these shortcomings, as clinicians may eschew addressing ambiguous topics. However, the unaddressed worries or concerns and unanswered questions, especially among historically marginalized women, highlighted areas where discussions could be improved. In many situations, it is appropriate for clinicians to not discuss breast density or supplemental screening depending on the woman’s overall risk profile or other pressing clinical concerns. Despite some reassuring findings regarding appropriate differential counseling by patient race and ethnicity, other results echo prior research documenting racial and ethnic variations in processes of care^23,24,25^ and provide new evidence of differential processes by patient literacy.

### Limitations

This study had several limitations. We were able to examine the content of discussions only among the women who had had such discussions, and these women differed in several ways from the national sample. Without clinical assessments of breast density, we were unable to determine whether breast density was a clinically significant or appropriate topic for clinicians to address in their discussions with women. Moreover, women’s self-reports of their discussions might be influenced by recall bias, and we did not have data on the length of time that elapsed between the mammography and these conversations. Although language barriers may have been an issue, without data on language we could not address this possibility in the analysis. In addition, we report results of a large number of statistical tests among correlated dependent variables, which warrants caution when interpreting significant findings. Future research efforts are needed to test the generalizability of observed associations, particularly those of smaller effect size. Care processes are not structured such that women are prompted to return to their clinician after mammographic screening (most see them before), so women reporting these discussions could have different health care use patterns relative to those who did not report conversations about breast density with their clinicians. They could have also been more concerned and sought out conversations, but our data did not allow us to examine these possibilities. Although the United States Preventive Services Task Force has not issued new guidelines for supplemental screening for women with dense breasts since this study was conducted, the American College of Radiology guidelines^26^ may have influenced counseling since then. Despite such limitations, women’s reports represent the most efficient method to assess the content of the conversations.

## Conclusions

Taken together, the results of this survey study provide some positive outcomes assessments when clinician-patient conversations about breast density do occur in that most patients report their clinicians are discussing potential breast cancer risk factors and mammography results and answering questions about breast density. However, these results also suggest potential areas for improvement as only a minority of our national sample reported such discussions, and when they occurred, there were limited conversations about other options for breast cancer screening, future breast cancer risk, or queries about patient worries or concerns about breast density. Efforts to improve cultural competency, shared decision-making, and counseling skills, especially when guidelines for care for women with dense breasts become more widespread, can potentially help close the remaining gaps in conversations about breast density.

## Notes

Supplement 1. **eAppendix.** Dense Breast Survey Omnibus Version

Supplement 2.
Data Sharing Statement
