# Supplementary material for: Sociodemographic Variations in Women’s Reports of Discussions With Clinicians About Breast Density
Source: JAMA Netw Open. 2023 Nov 27;6(11):e2344850. doi: 10.1001/jamanetworkopen.2023.44850 (PMC10682834; doi:10.1001/jamanetworkopen.2023.44850)
Supplement: Supplement 1. — eAppendix. Dense Breast Survey Omnibus Version [file jamanetwopen-e2344850-s001.pdf]

Supplementary Online Content

Kressin NR, Wormwood JB, Battaglia TA, Slanetz PJ, Gunn CM. Sociodemographic variations in women's reports of discussions with clinicians about breast density. *JAMA Netw Open.* 2023;6(11):e2344850. doi:10.1001/jamanetworkopen.2023.44850

eAppendix. Dense Breast Survey Omnibus Version

This supplementary material has been provided by the authors to give readers additional information about their work.

© 2023 Kressin NR et al. *JAMA Network Open.*

eAppendix.

DENSE BREAST SURVEY
OMNIBUS VERSION

*[**NOTE:** These questions are a subset of a larger survey. Only the survey items that pertain to the present report are included below. Thus, some of the skip patterns refer to items not included below, and the item numbers are not all sequential. The text below also includes instructions to the telephone interviewers who administered the survey (IN PARENTHESES AND CAPS).]*

(SSRS DEMO)
Z-7 What is your age?
(RECORD 2-DIGIT NUMBER)

____
99 Refused

(SSRS DEMO)
(ASK Z-7a IF Z-7=REFUSED)
Z-7a Could you please tell me if you are ...?
(PHONE ONLY)(READ LIST)

0 Under 18 (TERMINATE IF CELL)
1 18-29
2 30-39
3 40-49
4 50-64
5 65-76
6 77+
9 (DO NOT READ) Refused

(SSRS DEMO)
(ASK ALL)
(DO NOT READ) Enter sex of respondent

1 MALE
2 FEMALE

(IF FEMALE AGE 40-76 CONTINUE; ALL OTHERS SKIP TO NEXT INSERT)

BREAa1. Have you had a mammogram in the past 2 years?

1	Yes	(CONTINUE)
2	No	(SKIP TO NEXT INSERT)
9	(DO NOT READ) Refused	(SKIP TO NEXT INSERT)

© 2023 Kressin NR et al. *JAMA Network Open.*

BREAa2. Have you ever been told by a doctor or other healthcare provider that you have breast cancer?

(INTERVIEWER: IF RESPONDENT SAYS THEY WERE DIAGNOSED WITH DCIS (Ductal Carcinoma in Situ) OR LCIS (Lobular Carcinoma in Situ), CODE AS 'YES')

1	Yes	(SKIP TO NEXT INSERT)
2	No	(CONTINUE)
9	(DO NOT READ) Refused	(SKIP TO NEXT INSERT)

BREAa3. Before today, had you ever heard of the terms 'breast density' or 'dense breasts'?

1	Yes	(CONTINUE)
2	No	(SKIP TO NEXT INSERT)
8	(DO NOT READ) Don't know	(SKIP TO NEXT INSERT)
9	(DO NOT READ) Refused	(SKIP TO NEXT INSERT)

(SSRS DEMO)
S0. And to ask you the right questions, can you tell me what state do you live in?

[INSERT STATE LIST]

PROGRAMMER NOTE: IF FEMALE AGE 40-76 AND BREAa1=1 and BREAa2=2 and BREAa3=1, DBNQUAL=1; IF FEMALE AGE 40-76 AND BREAa1=2,9 or BREAa2=1,9 or BREAa3=2,8,9, DBNQUAL=2)

PROGRAMMER NOTE: IF FEMALE AGE 40-76 AND STATE= AK, AR, ID, IN, KS, ME, MS, MT, NH, ND, WV, or WY, DBNSTATE=0, IF FEMALE AGE 40-76 AND STATE= AL, AZ, CA, CO, CT, DE, FL, GA, HI, IL, IA, KY, LA, MD, MA, MI, MN, MO, NE, NV, NJ, NM, NY, NC, OH, OK, OR, PA, RI, SC, SD, TN, TX, UT, VT, VA, WA, or WI DBNSTATE=1)

(SSRS DEMO)
Z-10. Are you of Hispanic or Latino origin or descent?

1 Yes
2 No
8 Don't Know
9 Refused

© 2023 Kressin NR et al. *JAMA Network Open.*

(SSRS DEMO)

RT-01. Do you consider yourself white, black or African American, Asian, Native American, Pacific Islander, mixed race or some other race? (ENTER ONE ONLY)

(IF RESPONDENT SAYS HISPANIC ASK: Do you consider yourself a white Hispanic or a black Hispanic?)

(INTERVIEWER NOTE: CODE AS WHITE (1) OR BLACK (2). IF RESPONDENTS REFUSED TO PICK WHITE OR BLACK HISPANIC, RECORD HISPANIC AS "OTHER,"

If "other" say: "I'm not referring to your nationality. I just want to know if you consider yourself white or black."

If respondent won't pick one, then enter code for "OTHER"

1 White
2 Black or African American
3 Asian/Chinese/Japanese
4 Native American/American Indian/Alaska Native
5 Native Hawaiian and Other Pacific Islander
6 Mixed
0 Other (SPECIFY)__________
9 Refused

(SSRS DEMO)

Z-9. Is your total annual household income from all sources, and before taxes…? (READ LIST)

01	Less than $15,000 (12.5)		(SKIP TO Z-19a)
02	$15,000 but less than $25,000	(20)	(SKIP TO Z-19a)
03	$25,000 but less than $30,000	(27.5)	(SKIP TO Z-19a)
04	$30,000 but less than $40,000	(35)	(SKIP TO Z-19a)
05	$40,000 but less than $50,000	(45)	(SKIP TO Z-19a)
06	$50,000 but less than $75,000	(62.5)	(SKIP TO Z-19a)
07	$75,000 but less than $100,000, or		(87.5)
08	$100,000 and over		(125)
98	(DO NOT READ) Don't Know		(ASK Q.Z-9A)
99	(DO NOT READ) Refused		(ASK Q.Z-9A)

(SSRS DEMO)

Z-9a. Is your total annual household income from all sources and before taxes less than $50,000, $50,000 but less than $100,000, or $100,000 or more?

1 Less than $50,000 (35)
2 $50,000 but less than $100,000 (75)
3 $100,000 or more (125)
8 Don't Know
9 Refused

(SSRS DEMO)

© 2023 Kressin NR et al. *JAMA Network Open.*

Z-9b. Is that 100 to under 150,000, $150,000 to under $200,000, $200,000 to under $250,000, or $250,000 or more?

1 $100,000 to under $150,000 (125)
2 $150,000 to under $200,000 (175)
3 $200,000 to under $250,000 (225)
4 $250,000 or more (275)
8 (DO NOT READ) Don't Know
9 (DO NOT READ) Refused

(SSRS DEMO)
Z-19a. Would that be…?

(INSERT APPROPRIATE STUBS ONLY)

01 (IF Z-9=01) Less than $5,000
02 (IF Z-9=01) $5,000 to less than $10,000
03 (IF Z-9=01) $10,000 to less than $15,000
04 (IF Z-9=02) $15,000 to less than $20,000
05 (IF Z-9=02) $20,000 to less than $25,000
07 (IF Z-9=04) $30,000 to less than $35,000
08 (IF Z-9=04) $35,000 to less than $40,000
09 (IF Z-9=05) $40,000 to less than $45,000
10 (IF Z-9=05) $45,000 to less than $50,000
11 (IF Z-9=06) $50,000 to less than $55,000
12 (IF Z-9=06) $55,000 to less than $60,000
13 (IF Z-9=06) $60,000 to less than $65,000
14 (IF Z-9=06) $65,000 to less than $70,000
15 (IF Z-9=06) $70,000 to less than $75,000
98 (DO NOT READ) Don't Know
99 (DO NOT READ) Refused

© 2023 Kressin NR et al. *JAMA Network Open.*

(SSRS DEMO)

Z-8. What is the highest level of school you have completed or the highest degree you have received?

(DO NOT READ LIST)

(INTERVIEWER NOTE: Enter code 3-HS grad if Respondent completed training that did NOT count toward a degree)
(INTERVIEWER NOTE: Enter code 3-HS graduate if Respondent completed vocational, business, technical, or training courses after high school that did NOT count toward an associate degree from a college, community college or university (e.g., training for a certificate or an apprenticeship))

01 Less than high school (Grades 1-8 or no formal schooling) (OLD CODE 1)
02 High school incomplete (Grades 9-11 or Grade 12 with NO diploma) (OLD CODE 1)
03 High school graduate (Grade 12 with diploma or GED certificate) (OLD CODE 2)
04 Some college, no degree (includes community college) (OLD CODE 3)
05 Two-year associate degree from a college or university (OLD CODE 3)
06 Four-year college or university degree/Bachelor’s degree (e.g., BS, BA, AB) (OLD CODE 4)
07 Some postgraduate or professional schooling, no postgraduate degree (OLD CODE 5)
08 Postgraduate or professional degree, including master’s, doctorate, medical or law degree (e.g., MA, MS, PhD, MD, JD) (OLD CODE 5)
98 (DO NOT READ) Don’t Know
99 (DO NOT READ) Refused

© 2023 Kressin NR et al. *JAMA Network Open.*

(IF BREA9=1)

BREA11. I'd like to learn more about your most recent discussion with your doctor or other provider on the subject of breast density. When you last discussed breast density with your doctor or other provider did they also (INSERT ITEM)?

1 Yes
2 No
8 (DO NOT READ) Not sure
9 (DO NOT READ) Declined to answer

(SCRAMBLE ROTATE)

a. ask questions about your breast cancer risk, such as whether anyone in your family has had breast cancer, or if you have ever had a breast biopsy
b. ask about any worries or concerns you might have about your breast density
c. discuss your mammogram results with you
d. discuss other options for breast cancer screening, such as an MRI or Ultrasound
e. discuss your future risk of getting breast cancer

(IF BREA9=1)

BREA13. When you discussed breast density with your doctor or other provider, to what extent did your doctor or provider answer your questions about breast density? (READ LIST)

(ROTATE SCALE 1-5/5-1)

1 Completely
2 Mostly
3 Somewhat
4 A little
5 Not at all
8 (DO NOT READ) Not sure
9 (DO NOT READ) Declined to answer

CLINICAL RISK OF BREAST CANCER

BREA17. Have either your mother, sister, or daughter ever been diagnosed with breast cancer, or not?

1 Yes
2 No
8 (DO NOT READ) Not sure
9 (DO NOT READ) Declined to answer

BREA18. Have you ever had a breast biopsy, or not?

1 Yes
2 No
8 (DO NOT READ) Not sure
9 (DO NOT READ) Declined to answer

© 2023 Kressin NR et al. *JAMA Network Open.*

PERSONAL LITERACY LEVEL

BREA19. How often do you need to have someone help you when you read instructions, pamphlets, or other written materials from your doctor or pharmacy? (READ LIST)

(ROTATE 1-5/5-1)

1 Never
2 Rarely
3 Sometimes
4 Often
5 Always
8 (DO NOT READ) Not sure
9 (DO NOT READ) Declined to answer

© 2023 Kressin NR et al. *JAMA Network Open.*
