# Supplementary material for: Sociodemographic Variations in Women’s Reports of Discussions With Clinicians About Breast Density
Source: JAMA Netw Open. 2023 Nov 27;6(11):e2344850. doi: 10.1001/jamanetworkopen.2023.44850 (PMC10682834; doi:10.1001/jamanetworkopen.2023.44850)
Supplement: Supplement 2. — Data Sharing Statement [file jamanetwopen-e2344850-s002.pdf]

Data Sharing Statement

Kressin. Sociodemographic Variations in Women's Reports of Discussions With Clinicians About Breast Density. *JAMA Netw Open*. Published November 29, 2023.
doi:10.1001/jamanetworkopen.2023.44850

Data
Data available: No
